# “It just magically happened overnight!” – support for the digitalization of medical teaching provided by an interdisciplinary e-tutor team

**DOI:** 10.3205/zma001368

**Published:** 2020-12-03

**Authors:** Michael Abler, Regine Bachmaier, Birgit Hawelka, Stefan Prock, Silke Schworm, Anne-Kathrin Merz, Stephanie Keil

**Affiliations:** 1 Universität Regensburg, Fakultät für Medizin, Dekanat, Regensburg, Germany; 2 Universität Regensburg, Zentrum für Hochschul- und Wissenschaftsdidaktik (ZHW), Regensburg, Germany; 3 Universität Regensburg, Fakultät für Humanwissenschaften, Professur für Erziehungswissenschaft (Schwerpunkt Lernen mit visuellen Medien), Regensburg, Germany

**Keywords:** e-tutor, digital teaching, COVID-19, interdisciplinary team

## Abstract

**Background: **The forced and time-critical changeover to digital teaching and learning formats in the summer semester 2020 brought about numerous new challenges for the teaching staff of the Faculty of Medicine at the University of Regensburg. Didactic and personnel support of clinical lecturers for the preparation, creation, and supervision of digital teaching materials became necessary.

**Project description:** Since interdisciplinary teams seem to be superior in finding creative solutions, an interdisciplinary student e-tutor team was established at the Faculty of Medicine to support the digitalization of the range of courses. After their initial basic training the e-tutors had regular team meetings and internal mini-training sessions to ensure their continuous professional development. The e-tutors could be "requested" by clinical teaching staff and then accompanied the respective course preparation and implementation as required.

**Results and discussion: **Both clinical teachers and students perceived the student e-tutors’ support to be very positive. The e-tutors described the interdisciplinarity of the team as an important learning resource and their work as an exciting and instructive task.

**Conclusion and outlook: **Due to the positive experiences with the e-tutors, the faculty is striving to establish sustainable digital teaching and learning services in the coming semesters.

## Zusammenfassung

**Hintergrund: **Die erzwungene und zeitkritische Umstellung auf digitale Lehr-Lernformate im Sommersemester 2020 brachte für die Lehrenden der Fakultät für Medizin an der Universität Regensburg zahlreiche neue Herausforderungen mit sich. Didaktische und personelle Unterstützung der Lehrenden zur Vorbereitung, Erstellung und Betreuung digitaler Angebote wurde notwendig.

**Projektbeschreibung: **Da interdisziplinäre Teams in der kreativen Lösungsfindung überlegen scheinen, wurde ein interdisziplinäres studentisches E-Tutor*innen-Team an der Fakultät für Medizin etabliert, um die Digitalisierung des Lehrangebots zu unterstützen. Nach einem initialen Basistraining der E-Tutor*innen erfolgten zur kontinuierlichen Weiterentwicklung regelmäßige Teamtreffen und interne Mini-Schulungen. Die E-Tutor*innen konnten von den Lehrenden „angefordert“ werden und begleiteten dann bedarfsorientiert die jeweiligen Kursvorbereitungen und -durchführungen.

**Ergebnisse und Diskussion: **Die Unterstützung durch die studentischen E-Tutor*innen wurde von Lehrenden und Studierenden sehr positiv wahrgenommen. Die E-Tutor*innen beschreiben die Interdisziplinarität des Teams als wichtige Lernressource und ihre Tätigkeit als spannende und lehrreiche Aufgabe.

**Fazit und Ausblick: **Aufgrund der positiven Erfahrungen mit den E-Tutor*innen wird zur nachhaltigen Verankerung digitaler Lehr-Lernangebote an der Fakultät für die kommenden Semester eine Verstetigung dieser Unterstützungsleistung angestrebt.

## 1. Background

As a result of the SARS-CoV-2 pandemic, in the 2020 summer semester the Faculty of Medicine at the University of Regensburg (UR) had to switch teaching to predominantly digital formats. The technical and structural prerequisites for this were very good thanks to a Moodle-based learning management system and the provision of a powerful video conferencing system. Nevertheless, it soon became apparent that clinical teachers would need didactic and personnel support to prepare, create, and supervise digital teaching materials. In cooperation with the Centre for University and Academic Teaching at UR we developed and implemented a concept for an interdisciplinary e-tutor team. 

## 2. E-tutors

### 2.1. Interdisciplinary team

Diverse teams seem to be superior in developing creative and innovative solutions [1], [2]. When putting together the student assistant e-tutor team, we took care to integrate different relevant disciplines (medicine, education, media studies) as well as students from different semesters. The diverse composition of the team allowed us to combine medical and technical expertise with an understanding of teaching and learning theory, as well as technical know-how, thus providing the skills needed to support clinical teachers in creating and managing digital course materials.

#### 2.2. Training and support

The training and continuous support of the e-tutors was provided by

- the online course “Teaching with Digital Media – An Introduction”,
- the “First Aid Kit for Digital Teaching”, as well as
- regular team meetings and internal mini-training courses.

The online course “Teaching with Digital Media”, which was developed by the Chair of Educational Science (focus on learning with visual media) to support university teachers, offered an introduction to the design of digital teaching and learning materials that promote learning. 

The online portal “First Aid Kit for Digital Teaching”, which is maintained by the Centre for University and Academic Teaching, was used as another important source of information. The kit contains numerous suggestions, ideas, and assistance on how didactic elements (e.g. presentations with slides, discussions, group work) from classroom teaching can be adequately represented in digital settings. Since its release date in March 2020, the "First Aid Kit" has established itself as a central information and exchange portal for the design of digital teaching and has been expanded regularly.

A frequently used component of the “First Aid Kit” is a forum for university teachers to ask questions about technical and didactic problems. The community, which consists of experienced users from all faculties, usually answers very quickly and shares experiences and concepts. Thus, the “First Aid Kit” is not only an initial training element but also an ongoing support for the e-tutors.

In addition to the formal training offers mentioned above, regular team meetings were held as video conferences to discuss current challenges. We addressed the need for further training derived from these meetings independently and reciprocally in mini-training courses within the interdisciplinary team. For example, the tutors from non-medical subjects received an introduction to the content and progression of medical studies, whereas tutors from the field of educational sciences explained how Moodle courses can be structured to enhance learning, while tutors with special technical skills were available to explain the use of specific Moodle settings.

#### 2.3. Fields of application

The e-tutors primarily supported the clinical teachers in

- developing a learner friendly organization of the courses within the learning management system,
- communicating with students via the learning management system,
- improving existing and designing new learning units, as well as
- creating and using digital teaching materials.

Clinical teachers could “request” e-tutors from the Office of the Dean of Studies and were accompanied by them during course preparation, throughout the course or during the whole semester. The range of support provided varied greatly and depended on the previous didactic knowledge of the teaching staff as well as subject-specific requirements. In order to avoid a dependence of the clinical teachers on e-tutorial support, all support followed the principle of “help for self-help”. 

## 3. Results and discussion

Teaching staff and students alike perceived the support provided by e-tutors to be very positive. One clinical teacher was very grateful for the e-tutors’ support in restructuring her Moodle course, being amazed that “it just magically happened overnight”. The medical students were surprised by the didactic possibilities of the learning management system and would like to keep the new well-structured course design. The e-tutors themselves describe their work as exciting and instructive. They appreciate the cooperation in an interdisciplinary team, the mutual support, as well as the great personal responsibility. Of course, the e-tutors also had to deal with challenges. For example, they had to find a satisfactory balance between the teaching staff’s support expectations and their personal responsibility for their own digital teaching.

## 4. Conclusion and outlook

All in all, the e-tutor system has proven to be a great support for the clinical teachers as well as for teaching support units of the Faculty of Medicine. Interdisciplinarity and diversity strengthened cooperation and favoured mutual support. Guided by these consistently positive experiences, it is intended to permanently establish an interdisciplinary team of e-tutors.

## Competing interests

The authors declare that they have no competing interests.
